# Choosing Evolution over Extinction: Integrating Direct Patient Care Services and Value-Based Payment Models into the Community-Based Pharmacy Setting

**DOI:** 10.3390/pharmacy8030128

**Published:** 2020-07-24

**Authors:** Amanda R. Mercadante, Mai Yokota, Angela Hwang, Micah Hata, Anandi V. Law

**Affiliations:** College of Pharmacy, Western University of Health Sciences, Pomona, CA 91766, USA; amercadante@westernu.edu (A.R.M.); mai.yokota@westernu.edu (M.Y.); ara.hwang@westernu.edu (A.H.); mhata@westernu.edu (M.H.)

**Keywords:** community pharmacy, pharmacy benefit manager, healthcare, healthcare payment model, direct and indirect remuneration, pharmacy services, value-based payment, pharmacist role, COVID-19

## Abstract

The American healthcare payment model introduced Pharmacy Benefit Managers (PBMs) into a position of power that currently puts into question the state of the pharmacy profession, especially in the community field. Reimbursement plans had been designed to benefit all stakeholders and save patients money but have only been shown to increase costs for these involved parties. There exist unresolved gaps in care as a result of the healthcare structure and underutilized skills of trained pharmacists who do not have the federal means to provide clinical services. Four collaborative payment models have been proposed, offering methods to quell the monetary problems that exist and are predicted to continue with the closure of community pharmacies and sustained influence of PBMs. These models may additionally allow the expansion of pharmacy career paths and improve healthcare benefits for patients. With a reflective perspective on the healthcare structure and knowledge of positive impacts with the inclusion of pharmacists, solutions to payment challenges could present a progressive approach to an outdated system. The impact of the COVID-19 pandemic highlights a dependency on pharmacists and community settings. This outlook on pharmacists may persist and an established expansion of services could prove beneficial to all healthcare stakeholders.

## 1. Purpose

According to a study published in the *Journal of the American Medical Association* (*JAMA*), 1 in 8 pharmacies in the U.S. have closed down from 2009 to 2015 [1]. The California Society of Health-System Pharmacists (CSHP) presented that in the previous two years, an estimated 1 in 6 pharmacies had closed in the state of California alone. One substantial factor deemed responsible for these closures was the current reimbursement practice enforced by pharmacy benefit managers (PBMs). Pharmacists have noted that this reimbursement model is now antiquated, centered solely around the transactional dispensing of products. The pharmacy profession has reacted and expanded by transitioning its professionals into more clinical roles, however, the payment for clinical services remains underdeveloped. This paper aims to outline the issues with the current payment structure within the United States (US), its impact throughout the pharmacy profession, and to introduce adapted payment models that offer alternative approaches in the pharmacy profession; these include strategies from value-based payment (VBP) structures and integrated business models. 

## 2. Introduction

Reimbursement for dispensing medications has been the most common form of payment in community pharmacy practice. This heavy reliance on dispensing may have become detrimental to sustaining business; community pharmacies are no longer receiving full reimbursement for medications from PBMs. PBMs have perpetuated clawbacks and related fees with little to no regulation [2]. Community pharmacies have been challenged with retaining a profit and preserving business from receiving decreased reimbursements. 

Apart from the reimbursement issue, the lack of communication and transparency between patients, pharmacists, physicians, health plans, and PBMs add to the challenge of aligning a payment system. These models have a trickle-down effect on community pharmacies. Some of these unresolved gaps in healthcare management include the following:

1. Patient prescription information remains unknown to the health plan until the medication is filled and filed as a pharmacy claim or when the physician releases the information to the health plan directly from its office records.
2. There is no documented method that can adequately and inexpensively measure if patients are taking their medication exactly as directed. Adherence calculators have been utilized using pharmacy refill data; however, they do not accurately assess if patients are taking their medications correctly. Bioassays and digital chips in tablets are alternative examples of intrusive and expensive options.
3. Patients and primary care providers often do not know the cost of their medications. With numerous health plans and PBMs managing each formulary, prescribers are not aware of each patient’s preferred medication on formulary. This information can be provided to the patient, but they must call their health plan or related PBM directly. The patient also is not told the price of their medication until the pharmacy calculates the prescription through their computer system.
4. Upon receiving a prescription, a pharmacy is not always informed about the medication indication; exceptions exist in certain states where prescribers are required to write diagnosis codes directly on controlled prescriptions. Without a requirement for all states regarding all medications, the use for a medication can be unknown to both patient and pharmacist, especially if used for an off-label indication. There is a clear barrier within the process of informing the pharmacy what the prescriber’s office has intended the medication to treat.

In addition to prescription verification responsibilities in the community setting, pharmacists are required to offer counseling services to patients. The scope of practice for pharmacists expanded into patient education with the legislation of the Omnibus Budget Reconciliation Act of 1990 (OBRA-90). This act may have expanded pharmacist-provided work unrelated to dispensing, but it did not acknowledge or suggest any payment for pharmacists to provide this patient-centered and time-consuming service. Furthermore, the reimbursement for products to pharmacies comprises just a small dispensing fee. 

Pharmacists are now approved for increased responsibility through regulations in certain states. California implemented SB 493, which recognizes pharmacists as healthcare providers. Washington enacted SB 5557 which improves access to pharmacist services by requiring the inclusion of pharmacists in health insurance provider networks. Through HB 2028, Oregon grants pharmacists provider status and allows for the reimbursement of performed clinical services. Despite this forward movement in the profession, pharmacists are still separated from other healthcare professionals [such as Nurse Practitioners (NP) and Physician Assistants (PA)] because they do not have the federal provider status that enables billing for services directly to Centers of Medicare and Medicaid (CMS). 

## 3. History of Healthcare Payment Structure in the US

**Figure 1:**
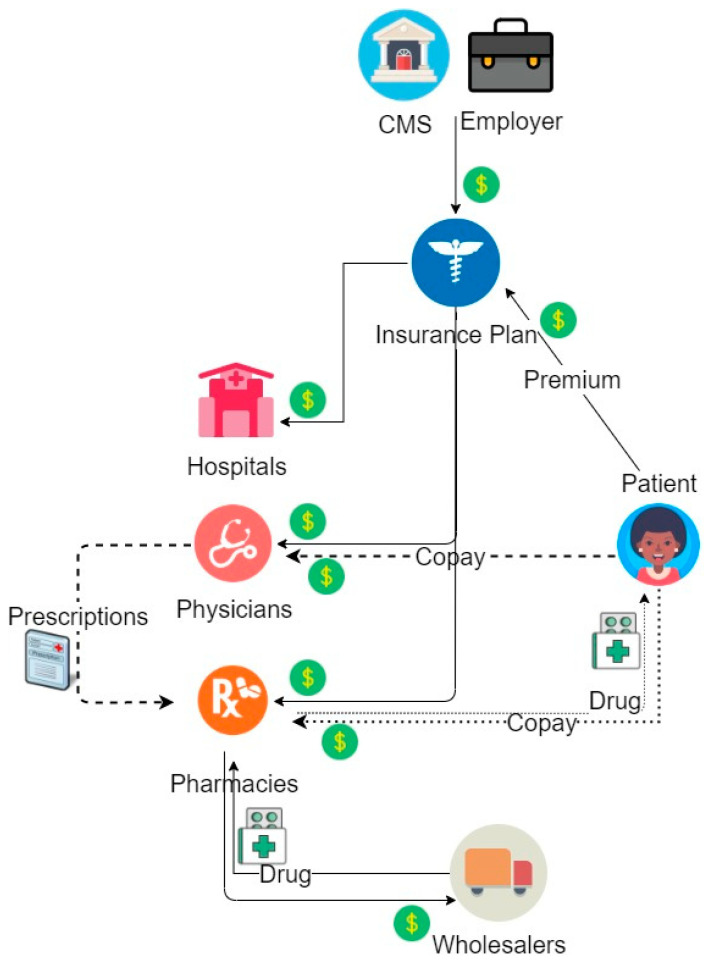
Reimbursement Model Prior to PBMs.

The current healthcare payment system consists of different stakeholders with varied interests in medicine and business. Prior to the rise of PBMs, cash flow started with CMS (previously “HCFA”) or specific employers who provided health benefits for employees/enrollees through contracts with “insurance plans,” later known as “health plans” (Figure 1). Insurance plans would then allocate payments to hospitals, medical offices, and pharmacies based on utilization and processed claims. Pharmacies paid their contracted wholesalers for providing their medications within the inventory while the insurance plans reimbursed pharmacies for drug costs and dispensing fees.

**Figure 2:**
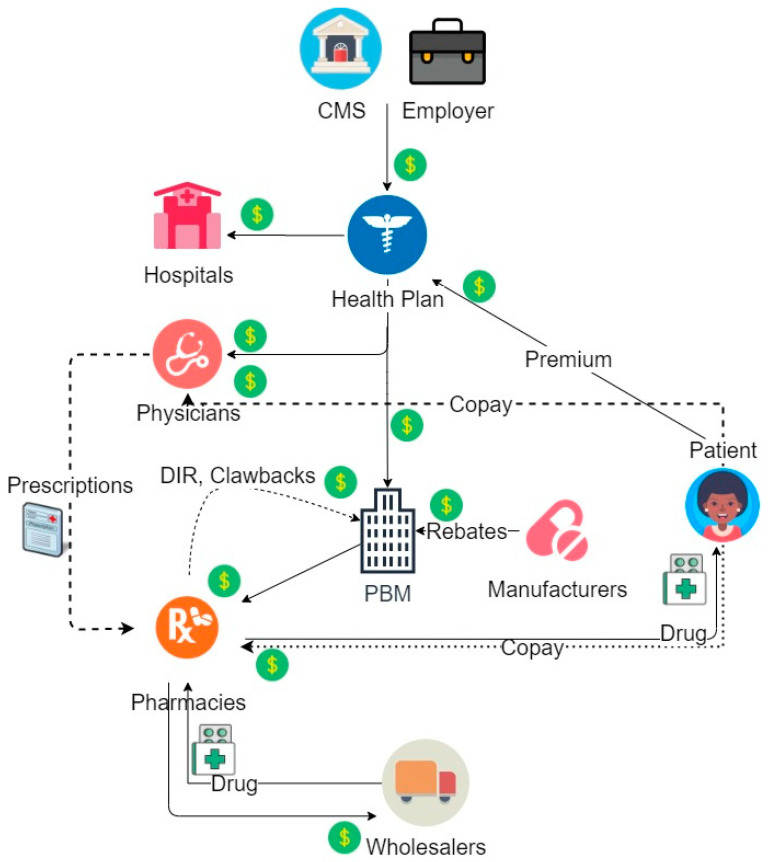
Addition of PBM to the Payment Structure.

As managed care prevalence increased and the amount of prescriptions grew in parallel, health plans found processing the large volume of claims to be unmanageable on their own. They chose to delegate this task to PBMs, thus introducing PBMs into the health payment system as a major player (Figure 2). PBMs were established in the late 1960s and were involved only in adjudicating pharmacy claims as the partner data warehouse. By the late 1980s, PBM roles began to evolve in conjunction with their responsibility for handling an increased volume of data [3]. PBMs began managing the medication formulary, choosing default medications in pharmacy inventories, and influencing which medications became the preferred choices by insurance plans. This role also established a new collaboration between the PBMs and pharmaceutical manufacturers who began leaning on PBMs to include their newly launched drugs into formularies. The incentive involved selecting drugs from a particular company as the preferred product when using a determined rebate system. Currently, PBMs do not only manage formularies, collect and analyze data, and adjudicate pharmacy claims, but are heavily responsible for the negotiation of drug prices, determining reimbursements, establishing prior authorization criteria, and forming pharmacy networks. Their income is ingrained in every piece of the model, reaching into health plans, pharmacies, and pharmaceutical companies. 

To illustrate the impactful presence of PBMs in this system, the three largest PBMs in the US cover more than 180 million lives (76% of the population) [4]. Furthermore, the biggest PBMs are included in Fortune 500′s top 10 companies every year [5]. PBMs have also become involved in vertical integration with health plans, chain pharmacies, specialty pharmacies, mail order pharmacies, and wholesalers. 

## 4. PBM Practices Impacting Pharmacies

There are a few pathways by which PBMs have impacted pharmacy reimbursement and patient care. One method involves routing patients into using mail order pharmacies that are owned or controlled by the PBMs. The incentive for patients is a lower copay for a 90-day supply (costing the price of only a 60-day supply) through mail order. Community pharmacies may offer their own 90-day supply savings along with health plans. Mail order not only decreases utilization of community pharmacies, but it also decreases the in-person interactions patients have with pharmacists. Many patients are greatly impacted by face-to-face counseling and require this intervention to maintain motivation to take their medications and to improve their long-term health care [6]. If mail order is to be implemented to satisfy both dispensing and educational needs, patient care and adherence need to be deliberately addressed to avoid potential consequences of distancing patients from pharmacists. In addition, depending on the health plan and PBM, the patient may be limited to a specific chain of pharmacies in order for their medication to be covered. This action may also limit the competitive ability for a patient to search for affordable medications and preferable individualized care from different pharmacies.

**Figure 3:**
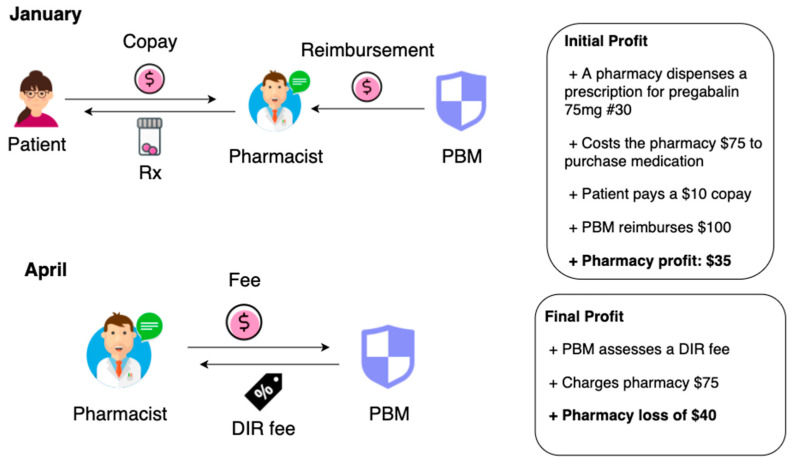
An example of the DIR fee process.

The mandated Direct and Indirect Remuneration (DIR) fees have also caused a ripple effect monetarily on community pharmacy settings and their patients. An example is illustrated in Figure 3. These fees were initiated when CMS created Medicare Part D in 2006 and were designed with the intention to pass along savings from the PBMs or Medicare Part D sponsors back to CMS [7]. The plan included returning savings back to Medicare beneficiaries by setting lower prices for medications. According to CMS, DIR fees include the following: discounts, chargebacks, rebates, cash discounts, free goods contingent on a purchase agreement, upfront payments, coupons, goods in kind, free or reduced-price services, grants, or other price concessions or similar benefits offered to some or all purchasers from any source, including manufacturers, pharmacies, enrollees, or any other person. DIR fees accounted for any savings that would serve to decrease the costs incurred by the Part D sponsor for the drug [8].

PBMs have been afforded the scope to enforce fees to commercial insurances in addition to Part D coverage. Community pharmacies are fined DIR fees under numerous different names such as network access fees, administration fees, or service fees. Oftentimes, these imposed fees are unknown to the pharmacy because there is no regulation, rule, or explanation on how and why these fees are determined. Additionally, these fees are charged retroactively which means that several months after dispensing the medication, the pharmacy is expected to make a payment the PBM demands (Figure 1) [9]. Legislation has allowed for zero oversight for any fees charged to pharmacies by PBMs [10]. This generous circumstance seems less of a coincidence when acknowledging that PBMs have paid USD 1.49 billion in lobbying during just the first three months of 2019 [11].

According to a CMS analysis, DIR fees have increased 1600% in the last five years and have amounted to USD 8.5 billion since 2013 [12]. The financial gain by PBMs has not been reflected in savings by Medicare Part D beneficiaries despite the originally advertised plan to save patients money. The true direction and distribution of these fees are unknown because there is no regulation to ensure that any cost savings the PBM collects ultimately gets passed onto the patients. In fact, Medicare patients continue to incur more out of pocket costs for prescription drugs with the existence of these fees [12].

## 5. Impact of Pharmacy Closures to the Profession

Although the aforementioned issues show a more immediate impact on community-based pharmacies, the effect of community pharmacy closures may reverberate throughout the profession. A few scenarios in affected settings and populations within the US are highlighted below.

### 5.1. Community Pharmacies

Community pharmacies are seeing a direct impact with decreased reimbursements and increased DIR fees. Moreover, the closure of these pharmacies leaves fewer job opportunities for graduating and practicing pharmacists and technicians. As a result, the prescription volume per staff in the remaining community pharmacies must increase to accommodate those patients forced out of their previous pharmacies. Increased prescription volume and stress for pharmacists could lead to errors of judgment, mistakes, and significant adverse drug events now seen in large-volume pharmacies [13].

### 5.2. Hospitals

With a reduction in the numbers of community pharmacies, patients may experience longer wait times at pharmacies, reduced access to medications, and face-time with pharmacists; subsequently, these may lead to more “medication misadventures.” Without the convenience of a community pharmacy to receive services, there may be increased reliance on hospital emergency departments and possibly more hospital admissions due to misadventures. This influx of patients could consequently increase the workflow for hospital staff and patient wait times in this setting as well. If patients are not being managed in community pharmacies, patient prioritization may need to be delegated to emergency visits, transitions of care, and other clinics. 

### 5.3. Patients

Patients, the ultimate recipients of health care, may be impacted the most by these aforementioned community pharmacy closures. A general reduction in the number of stores could result in less access for patients and decreased convenience; as stated previously, the consequences may include a dramatic increase in travel and wait times. Patients may also be motivated to call their primary care providers instead of the busy pharmacists, leading to an increased call burden at doctor offices. Some patients, especially those at higher risk, may require individualized attention or specialized care. Reduced access to this level of care due to fewer options could result in adverse patient outcomes. 

### 5.4. Ambulatory Care Pharmacies

With fewer community pharmacy options and potential increased burden on primary care providers, the need for ambulatory care clinics may drastically increase. Patients will need proper intervention and information to reach goal adherence while understanding their health needs. Pharmacists working in telehealth and Medication Therapy Management (MTM) may become the needed members of the patients’ individualized healthcare team if the community pharmacies are employed predominantly for dispensing purposes. 

### 5.5. Student Pharmacists

The direct impact on student pharmacists continues with fewer opportunities to gain experience and pursue a career in the community setting. The ability of rotation sites to provide practice experiences may be limited with the decreasing number of available pharmacies to precept students. Students will likely be shifted away from community pharmacy jobs and pushed to consider other career paths such as industry, research, and ambulatory care. The career prospects of pharmacy may change for many who envisioned a career in community pharmacy and the exponentially growing number of graduating students could face a more difficult task in paying off student loans. Additionally, the job market for pharmacists may not present paid positions for interns within their company if there are no open positions awaiting them as pharmacists. The pharmacies will not have the resources to train student interns that they cannot hire.

### 5.6. Academia

A combination of these previously described obstacles may lead to the decreased quantity and possibly quality of applicants for pharmacy schools. Recognition of a highly competitive job market with a Doctor of Pharmacy (Pharm.D.) degree may lead to a decreased applicant pool. Rotation sites may also be burdened with higher student ratios per preceptor if other sites are no longer available. The education of students may need to adapt to the opportunities that await graduates; classes focusing on skills for community pharmacy may take a backseat to allow for further education regarding industry, residential, and research pharmacy skills. This awareness of the professional climate could influence pharmacy programs to change certain aspects of their curriculum and present new elective options.

## 6. Changing Roles of Community Pharmacists 

In response to the decrease in reimbursement for dispensing medications, community pharmacists have evolved to find solutions and provide additional services. A list of newly initiated programs is shown in Table 1. One initiative includes retail pharmacy chains partnering with general practitioners (e.g., physicians or nurse practitioners) to set up clinics within pharmacies and see patients for acute conditions. Newer online startup pharmacies have been offering free delivery services similar to mail order pharmacies. Certain pharmacy chains have also offered lower cash prices on a list of generic medications (e.g., metformin, lisinopril) to patients who do not carry insurance. One pharmacy has teamed up with a PBM, promising no DIR fees, no clawbacks, and no hidden charges; utilization of a code suffices as a coupon price for partnering pharmacies and do not require insurance use. The patient can also elect to have the prescription filled online with free delivery for medications. Some pharmacies have adjusted their medication stock, no longer ordering those medications for which they will not be reimbursed or have previously lost money from DIR fees; these pharmacies direct patients to other pharmacies that may carry the medication on their formulary.

Other strategies community pharmacies have implemented include the addition of clinical services to their dispensing model. AB 1114 in California encourages pharmacists to provide certifiable services such as furnishing travel medications and birth control. These services can be paid through Medi-Cal when provided by the certified pharmacists [14]. Another currently billable service performed by pharmacists includes MTM. While MTM can be reimbursed by a third-party insurance company, the monetary amount may not be equivalent to the investment of time pharmacists require to complete a single MTM encounter. Infrequently, private insurance companies will pay for clinical services, but the process for compensation can prove cumbersome, requiring individual contracts with every individual pharmacy location providing MTMs. Ultimately, pharmacists cannot directly bill Medicare for services such as chronic care management or transitions of care because they are not federally recognized as healthcare providers. A collaborative practice agreement (CPA) between a pharmacist and physician is currently the most effective way for a pharmacist to provide specific clinical services; their respective billing codes may allow for payment of those services. “Incident-to” billing is required for any outpatient services that a pharmacist is approved to provide after satisfying several requirements: incident-to billing can be conducted by pharmacists only in conjunction with a Medicare-approved provider and when in restricted settings (in the same office suite, which usually means not within a community pharmacy); this billing has limits the reimbursement. In addition, the patient must first be seen by a physician; the physician must then authorize the use of the patient’s medical record, directly supervise the pharmacist, and remain readily available. This provision of care is also paid as fee-for-service, showing no monetary regard for health outcomes. 

## 7. Proposed Models for Pharmacy Payment

The authors utilized a variety of methods to develop the following payment models. A literature search identified studies using electronic databases PubMed and International Pharmaceutical Abstracts (IPA). The search was not specifically limited to any time period but did focus on the most recent US data regarding payment models. The following search terms were utilized: health care payment model, pharmacy model, value-based payment, fee for service, community pharmacy role, pharmacist services, community pharmacy services. A supplementary approach utilizing media (Google search engine) and pharmacy newsletters and articles (e.g., American Pharmacists Association (APhA), California Pharmacists Association (CPhA), Pharmacy Times) presented documented examples of business attempts to resolve payment issues and more current descriptions of actions taken by the pharmacy profession that were not found in the electronic databases. Eligibility assessment for relevant articles was conducted independently by the authors and any disagreements were discussed during preliminary development of adapted payment models. In addition, the authors prepared and led a presentation on the gaps in healthcare systems and issues with reimbursement for pharmacists in community-based pharmacies to practicing faculty at a college of pharmacy. Based on professional input and discussion with an expert panel of professional society advocacy groups, four gaps were identified. Finally, the authors conducted expert interviews between February and April 2020 with four independent pharmacy owners and four health plan administrators. These discussions introduced the authors’ proposed models to the professionals and allowed for the collection of valuable stakeholder insight regarding various payment models, opinions on proposed payment models, and challenges and opportunities within each model. After assessing the recorded interviews, four proposed models were filtered, refined, then selected for this paper. Summaries of the completed models with their respective pros and cons are listed in Table 2. 

### 7.1. Model 1: Pharmacist Attached to Primary Care Physician (PCP) Offices

Primary care providers have expanded from medical doctors to include NPs and PAs. These providers have the authority to diagnose and prescribe medications (based on state laws and scope of practice). In this model, a pharmacist would cohabitate the same office as the PCP, allowing for a one-stop appointment where a patient can have designated time with both professionals.

While the pharmacist would be available to directly counsel patients and work with the physician, the dispensing of medications would be kept separate. This option would allow patients to utilize mail order or pick up medications at a pharmacy of their choice or at the behest of the health plan. The compensation for this office-based pharmacist would also be dependent on clinical services. While this model is not novel and has been implemented occasionally in the past, the payment structure may provide new elements. This model has also been proposed by the 2019–2020 President of the American Association of Colleges of Pharmacy (AACP); “his bold aim is that by 2025, 50 percent of primary care medical practices will have integrated comprehensive medication management (CMM) services into their care model, and those services will be delivered by pharmacists [15].” 

While MTM services can be billed per patient, the entire healthcare group responsible for taking care of the patient would benefit from collaborative practice; the co-pay for the visit would not only go towards the physician, but the pharmacist as well. While utilizing the idea put forward through collaborative practice agreements and incident-to or value-based payments, these services would be utilized more efficiently and more frequently because of the synergized setup. Through this team model, the physicians would be able to focus more on the examinations and diagnostic services while the pharmacist would contribute through medication-related and lifestyle counseling as well as educating the patient about their adherence goals. Moreover, pharmacist collaboration could help physicians with quality measures such as Healthcare Effectiveness Data and Information Set (HEDIS), Merit-Based Incentive Payment (MIPS), and Medicare Access and CHIP Reauthorization Act (MACRA) for increased compensation. Delegated services would provide a more comprehensive experience and allow for direct communication and organization of patients’ medical and lifestyle plans. There would no longer be any gaps or missing information between these professionals concerning patients’ diagnoses or medical records.

One study demonstrated that MIPS was able to “reduce administrative burdens, protect practices serving vulnerable populations and improve communication between program administrators and primacy care providers (PCPs),” but was limited by the potential movement of clinical resources away from patient-centered care and decreased patient and clinician satisfaction [16]. This study explained that the model would need to demonstrate simplicity in design and allow practical adaptability to prove its benefits outweighed its challenges. PCP interviews revealed that burdens of time and participation, risk of penalties, and over-complication of the administration of an adapted program would be great disadvantages to incorporating a value-based model [16]. Similar issues may present themselves in this model as well. Additionally, incident-to billing may limit this model due to its stringent requirements, hence other value-based payments may be preferable to health care providers and stakeholders. 

### 7.2. Model 2: Transparency Payment Model

As previously stated, there exists a gap in communication between a health plan and patient medication records. Healthcare is the only industry where the ‘consumer’ is uninformed of the price for the product prior to purchase other than the co-pay or cost share. This transactional irregularity has resulted in ‘Surprise Billing’ for patients who receive multiple and excessive bills from doctors and hospitals. A bill has been proposed to protect patents from receiving large bills called the Surprise Billing Legislation, but at the time of this writing, has not yet been passed [17].

In this proposed payment model, patients would have the option to pay directly with cash or through insurance. If patients have access to the drug price, they can control how and where they would pay. Previously, pharmacies were concerned about insurance-only billing due to reimbursement by the PBM. In the proposed scenario, pharmacies would be free to offer patients the best cash price. Patients would have the option to pay cash price for their prescriptions and save their insurance dollars for more expensive medications (as insurance was intended to do). This would benefit the health plan payout because they would not be charged for all prescriptions. 

The transparency model could be optimized with a medication price-tracker for patients. A similar model has been established in certain pharmacies where patients are able to price shop prior to switching to that pharmacy.

The transparency model shares similarities with the value-based benefit design implemented by some plan sponsors or PBMs; these plans negotiate a lower copay for high value prescriptions (e.g., statins) based upon established patient health outcomes or pre-set financial incentives [18]. The challenge in translating this model to pharmacies is the responsibility of tracking patient outcomes that are conducted by plans or PBMs. Additionally, this design benefits the larger volume stores that have a bigger bargaining power with wholesalers.

### 7.3. Model 3: Accountable Care Organization (ACO) Plus Patient Model (ACOPP)

An ACO-based model with the addition of the patient (ACOPP) is a shared risk value-based system that distributes accountability for inputs and outcomes among vested parties. Until now, ACOs have included hospitals, physician offices, nurses, and other healthcare professionals that have demonstrated success in cost savings and health outcomes for high-risk patients. However, these models have not included proactive roles involving patients. 

The authors propose an integrated model with physician, pharmacist, and patient in a modified ACO. Accountability remains the focus: all parties involved would have shared risk that could result in payment benefits such as lowered copays for patients and monetary rewards for medical providers and pharmacists. If conditions are not met or value is not achieved, the risk could include withholding of payment benefits. The health plan takes risk by offering these payment benefits if goals are achieved, which would be offset by the long-term cost savings associated with improved medical outcomes and a reduction in high cost visits such as hospitalizations and emergency care. For example, a health plan could offer lower premiums to patients for incentivized measures such as documented yearly lab work if required by prescriber and yearly overall documented proportion of days covered (PDC) ≥0.80 for chronic medications. To include the risk aspect, health plans could require higher premiums or withhold low-cost benefits if the patient does not maintain an appointment with a general practitioner and have lab work at least every 2 years, or if the patient’s documented annual PDC falls below a specified number. Payments could be determined on an annual basis to ensure adequate time for goals to be attained.

The major challenge in this model is the full participation of health plans and patients. Without all parties involved and actively collaborating, the shared risk could be greater than the shared benefit. Patients have been acknowledged as passive receivers of healthcare. This model would require an aspect of motivation; patients would need to be invested and take an active role in their healthcare. Accordingly, patients taking responsibility and being proactive could help their long-term outcomes and positively affect their perspective and “locus of control” when actively involved as a member of the team. Another challenge exists with the downside risk agreements that are necessary in an ACO-like model. Downside risks are designated amounts of loss that could occur in an industry or company if the market or item values decline. For example, a healthcare system would be responsible for downside risk if the care exceeds the thresholds set for finances and clinical services. This system provides innate accountability for providers and payers when delivering patient care. The 2018 Annual ACO Survey showed that there is still much disagreement with the adoption of contracts with downside risk and how to compel health care organizations to be more invested in the management of public health [19]. Proper contracting and further discussions with Medicare and commercial groups would be essential to ensure the success of this type of model.

### 7.4. Model 4: Pharmacist Network

Healthcare professionals have utilized a network model in order to best serve communities and provide them with a provider that is available and convenient to their location. Toll free numbers allow patients to connect with and seek the help they need when there is a network of professionals involved. Community pharmacies serve similar purposes of convenience but are limited to dispensing duties with a small amount of time for counseling per patient. 

A pharmacy network model could be implemented to connect pharmacists with a variety of clinical services (that are not provided in the community pharmacies) directly to the patients in need. This model would necessitate a call center that has a list of verified pharmacists available for temporary or contract positions. Independent pharmacies or companies looking for contract pharmacists for ambulatory or clinical care services would be able to connect with a pharmacist for a negotiated period of time. Patients would be able to contact the network to get in touch with a pharmacist for medication-related questions and services. The primary advantages of this model would include addressing the multibillion-dollar medication nonadherence and medication misadventure issues while also highlighting the integral role of the pharmacist. This network would also allow pharmacists currently in different practices who want to transition into clinical roles to receive training and find work opportunities. With the increasing number of graduate pharmacists in the US, these opportunities could provide employment pathways for many new professionals [20]. Patients would have appropriate health care from a clinical pharmacist just one phone call away. Community pharmacy networks (i.e., CPESN) and companies currently exist that provide services for either patients or healthcare systems such as MTM or medication risk mitigation management, but their focus is on specific services or locations. This network model is broad and would include retail, hospital, and ambulatory care pharmacists.

Starting a pharmacy network would require a large amount of funding or investment to offset the salaries, benefits, and incentives for pharmacists to join the network. Marketing would also be needed to increase awareness of the services and to change patient expectations of pharmacists.

## 8. The Impact of COVID

The scenarios resulting in the healthcare industry as a result of the COVID-19 pandemic have shaped a new outlook for pharmacists that may persist after solutions enable us to address the impact of the disease. The Ohio Department of Health, for example, has created a checklist and new standards for pharmacists; these dictate that the pharmacist is the major influence on everyday patients and will be establishing a precedent of “calmness, knowledge of procedures, and safe protocols” within the pharmacy in addition to the normal duties of counseling, dispensing, and maintaining business operations [21]. The current situation has established great attention on pharmacists and their broadened roles in preventative health. Physician offices have closed or reduced hours, reserving appointments for emergencies or high-risk patients, while pharmacies have stayed open. In an effort to increase the span of the healthcare providers, states (e.g., NY, PN, MA) have fast-tracked the abilities of new medical doctors and nurses. Pharmacists have been allowed increased services such as conducting health and wellness tests, managing chronic diseases, performing medication management, and administering immunizations [22]. Certain retail pharmacies utilize pharmacists to test patients for COVID-19, a previously unprecedented task in a community pharmacy [23].

If a vaccine becomes available for COVID-19, as expected before the end of 2020, it is likely that community pharmacists will be the major group (if not the only medical group) responsible for administering and counseling patients regarding expectations of the vaccine. Patient counseling and education could become even more important and time-consuming for pharmacists in this setting if more than one vaccine is approved for administration. 

During this time, in a temporarily expanded model, pharmacists have been able to represent their specialized skills and abilities beyond their usual duties. These examples display that pharmacists can fill needed gaps in care through providing clinical services. If supported in the future by provider status and billing for services, the adaptation of a new payment model could properly fit the demands of patients and the healthcare system. 

## 9. Conclusions

The current state of outpatient community-based pharmacies presents many challenges for pharmacists and other stakeholders in US healthcare. The involvement of PBMs has altered the structure of healthcare and allowed for their own benefit at a direct cost to community pharmacies. The current methods enforced by PBMs with limited governmental regulation will continue to push community pharmacies out of business if they remain in their current position. The challenges with this payment structure and abilities of pharmacists could be viewed as an opportunity to further the practice of pharmacy and influence the healthcare field. The authors propose various payment models that could offer solutions where pharmacists could better control their professional status and profit margins. Research and implementation are required to demonstrate the impact of these models. While the models are not necessarily mutually exclusive in ideology, there would be value in testing these with a design that allows comparison to the current payment structure and examination of the outcomes. 

## Figures and Tables

**Table 1:** Community Pharmacy Program Initiatives.

Added Pharmacy Services	Companies
Free delivery	Online, chain, independent pharmacies
Medical clinics	Chain stores
Clinical services: oral contraception, travel immunizations, nicotine addiction treatment	Chain, independent
Low cash prices for generic medications for uninsured patients (metformin, lisinopril, etc.)	Chain stores
Coupons	Rx coupon websites, manufacturers
Coupon with PBM	Online pharmacy

**Table 2:** Summarized Attributes from the Proposed Payment Models.

Payment Model	Pros	Cons
Pharmacist in PCP Office	Dispensing is separate Direct cooperation and collaboration between pharmacists and physicians Comprehensive care for patients in a single setting	Patients must pick up prescriptions from a pharmacy or mail-order Scheduling for time with both PCP and pharmacist could increase patient wait-time if not handled appropriately Potential burdens of time, participation, risk of penalties, and over-complication of administration
Transparency Model	The drug prices are known to the patient and established by the pharmacy Drug prices can be competitive between independent pharmacies Potential to completely eliminate need for a PBM Most advantageous for patients who are uninsured or underinsured for prescription drugs	May limit the number of drugs available to be dispensed if high-cost drugs are no longer bought by the pharmacy Wholesalers may sell drugs at lower prices to chain stores with large volume of medications, discriminating per store-volume May not be able to accept insurance coverage Additional responsibility to track all medications independent of insurance use
Shared Risk VBP (ACOPP)	Shares the responsibility and burden of health care with patients May work easily in already established ACOs with contracts with health plans Promotes collaboration and communication between providers and pharmacists	Success is dependent on buy-in from all parties involved The risk aspect of the model may be challenging for health plans to implement in patients on public programs (e.g., Medi-Cal)
Pharmacist Network	Employers are able to utilize qualified pharmacists on an as-needed basis Allows pharmacists who want to transition into clinical roles to find opportunities of work in a specified region Patients can easily access a pharmacist convenient to them and specific for their health needs	Requires a large investment or funding source for initial startup fees Advertising the usefulness of the clinical pharmacists and why patients would call the network could prove challenging, especially short-term
